# Direct synthesis of high quantum yield lead‐free CsCu_2_I_3_ powder in water and its application in yellow LED

**DOI:** 10.1002/EXP.20240004

**Published:** 2024-07-18

**Authors:** Heng Guo, Linlin Shi, Zengliang Shi, Yue He, Yizhi Zhu

**Affiliations:** ^1^ Department of Science Taiyuan Institute of Technology Taiyuan People's Republic of China; ^2^ College of Electronic Information and Optical Engineering Taiyuan University of Technology Taiyuan People's Republic of China; ^3^ State Key Laboratory of Digital Medical Engineering School of Electronic Science and Engineering Southeast University Nanjing Jiangsu People's Republic of China; ^4^ Microcellular Plastics Manufacturing Laboratory Department of Mechanical and Industrial Engineering University of Toronto Toronto Ontario Canada

**Keywords:** 2‐methylimidazole, CsCu_2_I_3_, quantum yield, stability

## Abstract

Yellow light‐emitting diodes (LEDs) with a wavelength of 570–590 nm can reduce the excitability of peripheral nerves and the sensitivity of the skin, stimulate collagen synthesis, and tighten the skin, which plays an important role in skin rejuvenation. In general, commercial LEDs are made of phosphor excited by ultraviolet chips. It is very important for the development of yellow light emitters with high luminous efficiency, good stability, and environmental protection. For the first time, a simple organic structural unit (2‐methylimidazole, 2‐MIM) was used to collect a mixture of two metal precursors (CsI and CuI) and successfully synthesized an all‐inorganic lead‐free yellow light CsCu_2_I_3_ powder in water. The prepared CsCu_2_I_3_ powder exhibited excellent optical properties and considerable stability. Finally, a phosphor‐converted LED (pc‐LED) device was fabricated via the CsCu_2_I_3_ phosphor coated on a 310 nm ultraviolet chip. The pc‐LED device's electroluminescence spectra may be a good fit for the blood's absorption regions. Therefore, this work provides a facile method for the synthesis of novel lead‐free metal halide CsCu_2_I_3_ powder in eco‐friendly solvents. In addition, the stable and efficient CsCu_2_I_3_ powder shows promising exciting potential applications in photoluminescence and phototherapy fields.

## Graphical abstract

For the first time, a simple organic structural unit (2‐methylimidazole, 2‐MIM) is used to collect a mixture of two metal precursors (CsI and CuI) and successfully synthesize an all‐inorganic lead‐free yellow light CsCu_2_I_3_ powder in water. The stable and efficient CsCu_2_I_3_ powder shows promising exciting potential applications in photoluminescence and phototherapy fields.

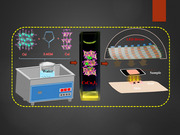

## 1 INTRODUCTION

The rapid development of light‐emitting diodes (LEDs) as a revolutionary light source, represented by great brightness and low cost, has seen substantial advancement across business, manufacturing, medical, and light therapy sectors.^[^ ^1^ ^]^ Yellow light, as a warm color light, has the advantages of uniform spectrum distribution, stable brightness, and no glare, which is widely needed by the lighting industry. At the same time, LEDs having an emission wavelength ranging from 570 to 590 nm well match the absorption band of blood vessels and can penetrate the skin between 0.5 and 2 mm.^[^ ^2^, ^3^ ^]^ Hence, photo‐aging, stress relief, and using YLEDs as a supplement to laser therapy are the main areas of application for these devices.^[^ ^4^, ^5^, ^6^, ^7^ ^]^ Therefore, it is of great significance to develop yellow light‐emitting materials with good stability and high luminous efficiency.

Over the last two decades, lead halide perovskite nanocrystals and quantum dots with excellent optical properties have received considerable attention in photoluminescence material research. Lead‐based halide perovskite with the ABX_3_ composition (A: CH (NH_2_)_2_^+^, CH_3_NH_3_^+^ (MA), Cs^+^, B: Pb^2+^ and X: I^−^, Cl^−^, Br^−^) have been known since the 1950s.^[^ ^8^, ^9^, ^10^, ^11^ ^]^ Although they have exceptional optical characteristics, such as strong light absorption, narrow emission spectrum, and adjustable emission wavelength. However, the Pb has meager air stability and high toxicity, which hamper their deployment on large‐scale commercial prospects. Therefore, developing environmentally friendly lead‐free perovskite luminescent materials has become a research hotspot. A simple method is to replace Pb^2+^ with Sn^2+^ or Ge^2+^, which is a less toxic element of the same family.^[^ ^12^ ^]^ Unfortunately, the stability and optical properties of the obtained compound CsBX_3_ (B = Sn^2+^ and Ge^2+^) are lower than those of lead‐based perovskites, due to the easy oxidation of Sn^2+^ and Ge^2+^ to the corresponding 4+ states.^[^ ^13^, ^14^, ^15^ ^]^ An alternative crucial approach involves substituting Pb^2+^ ions with one monovalent (M′) and one trivalent (M′′) cation to create M′ and M′′ octahedra, resulting in the formation of a 3D double perovskite structure A_2_M′M′′X_6_, such as Cs_2_AgBiBr_6_,^[^ ^16^ ^]^ Cs_2_AgInCl_6_,^[^ ^17^ ^]^ (CH_3_NH_3_)_2_AgBiBr_6_,^[^ ^18^ ^]^ and Cs_2_AgTlBr_6_.^[^ ^19^ ^]^ These double perovskites have wide or indirect band gaps, which results in low PLQYs, which hinders their application in high‐brightness LEDs. In addition, the enclosure of heavy/noble metal cations (Ag^+^, In^3+^, and Bi^3+^) may be detrimental to lead‐free double perovskite fabrication. Recently, the use of copper (I) to completely replace Pb in the production of all‐inorganic lead‐free metal halide emitters, such as Cs_3_Cu_2_X_5_ and CsCu_2_X_3_,^[^ ^20^, ^21^, ^22^ ^]^ has attracted the attention of researchers. Because it has the following advantages: (i) the non‐toxicity element Cu is friendly to the natural environment, and (ii) it has abundant reserves on Earth. However, the reported synthesis of copper‐based perovskite is accomplished by anti‐solvent vapor saturation, vertical Bridgman method, and hot injection. These methods are in need of high temperature or vacuum, which is cumbersome for the operation of waste energy and is not conducive to environmental protection. Therefore, the simple and environmentally friendly synthesis of Cu halide luminescent materials, which may simultaneously achieve high PLQY and good stability is interesting.

This study focused on the sub‐family of CsCu_2_X_3_, we introduced a facile approach and successfully synthesized CsCu_2_I_3_ powder in water for the first time by replacing Pb^2+^ with Cu^+^. The morphology and luminescence spectrum of the perovskite were characterized. The PLQY of CsCu_2_I_3_ is as high as 49.62%, and it exhibited good stability in air and water under UV light. By using the prepared metal halide CsCu_2_I_3_ as a phosphor to produce a UV‐pumped LED, bright yellow light was emitted, indicating that the LED is expected to be used for yellow light therapy.

## 2 EXPERIMENTAL DETAILS

### 2.1 CsCu_2_I_3_ powder preparation

CuI (98.0%), CsI (99.5%), and 2‐methylimidazole (98%) were obtained from Aladdin. All chemicals were utilized in their received state without undergoing any additional purification steps. At first, 0.5196 g of CsI, 1 g of 2‐methyl‐imidazole (2‐MIM), and 0.7618 g of CuI were added to one pot, and then added 5 mL water. Upon magnetic stirring for 5 min, the reaction mixture promptly transitioned to a brown color. Subsequently, the mixture was sonicated 40 min, and the sample color changed to milky white. Finally, the CsCu_2_I_3_ sample was cleaned by using water and collected via centrifugation.

### 2.2 Characterization

The X‐ray diffraction analysis (XRD) analysis was carried out on Ultima IV with Cu Kα radiation (*λ* = 1.54 Å), diffraction patterns were scanned within an angular range of 10°–70°. A Carl Zeiss Ultra Plus scanning electron microscope fitted with X‐ray energy‐dispersive spectroscopy (EDS) was used for elemental analysis and scanning electron microscopy (SEM). Vienna ab initio Simulation Package (VASP) was used to perform density functional theory‐based first‐principle calculations utilizing the plane‐wave pseudopotential technique. The General Structure Analysis System (GSAS) program assisted in the refinement of the structural characteristics of the CsCu_2_I_3_ sample by the application of the Rietveld method. A continuous‐wave Xenon lamp was used as the excitation source in a spectrofluorometer (F4600) to acquire photoluminescence (PL) and photoluminescence excitation (PLE) spectra. Using an integrating sphere‐equipped fluorescence spectrometer (Fluorolog 3‐TCSPC), the photoluminescence quantum yields (PLQY) of the samples were determined. The Fluorolog 3‐TCSPC spectrometer's time‐correlated single‐photon counting technology was used to record decay curves. Every measurement that was done above at room temperature. A thermostat was utilized to regulate temperature‐dependent photoluminescence, and liquid nitrogen was utilized to attain low temperatures. A spectrophotometer fitted with a photomultiplier tube detection device was used to record emission spectra. The excitation light source was a 325 nm femtosecond laser, and an automatic tuning temperature controller (Linkon) was used to modify the temperature in increments of 10 K for each spectrum that was recorded. The temperature range was 80–300 K.

### 2.3 Fabrication of LED device

Initially, CsCu_2_I_3_ powder was thoroughly mixed with silicone to have a uniform distribution. Then, the LED device was fabricated by coating the above mixture on a 310‐nm UV chip surface. The integrating sphere spectroradiometer system (HP9000) was employed to investigate the photoelectric properties of as‐fabricated LED device (operated at 3 V and 20 mA driven current).

## 3 RESULTS AND DISCUSSION

**FIGURE 1:**
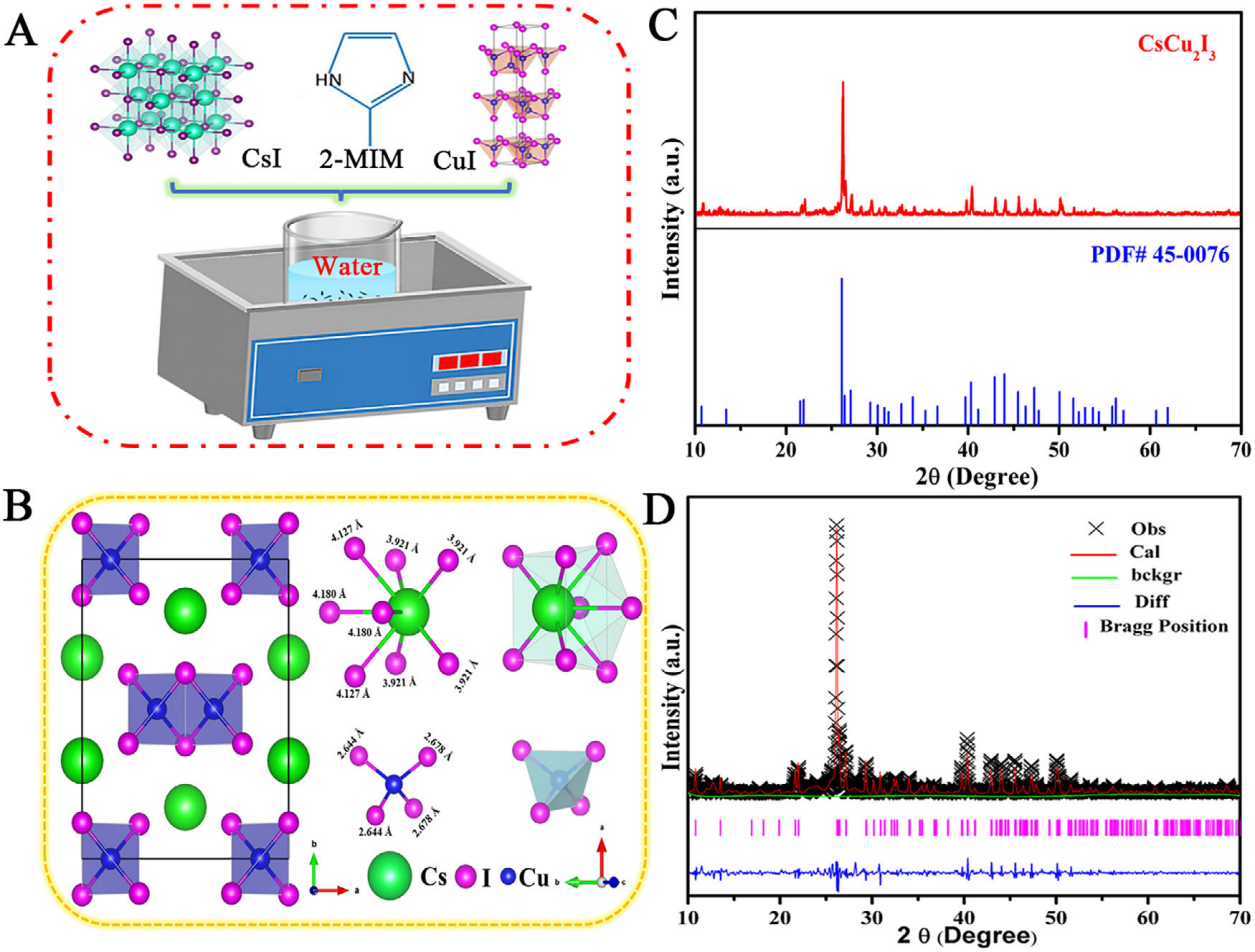
Architecture and characterization of CsCu_2_I_3_ powder. (A) Scheme for preparation of the CsCu_2_I_3_ powder. (B) Structure of CsCu_2_I_3_ powder with [CuI_4_]^3−^ polyhedron displayed in purple color, and Cs^+^ cations in green. (C) XRD pattern of the sample (top), compared with the orthorhombic CsCu_2_I_3_ at the bottom (JCPDS code 45–0076). (D) Rietveld refinement of CsCu_2_I_3_ powder.

Herein, we report a simple method for synthesizing CsCu_2_I_3_ powder by ultrasonic treatment with a green solvent (water) throughout the whole process. To efficiently synthesize CsCu_2_I_3_ powder in water, simple organic 2‐MIM was used as a reaction adjuvant to promote its synthesis. Figure 1A illustrates the synthetic process. In short, CsI, 2‐MIM, and CuI were added to one pot, and then added deionized water. Finally, the powder is obtained by ultrasonic and centrifugation. Detailed operation is shown in the experiment section Notably, no high temperatures or noble gas shielding is required; the entire synthesis process takes place in the presence of air and at ambient temperature. This greatly reduces the preparation cost and complexity of preparation. The synthesized CsCu_2_I_3_ crystallizes in an orthogonal Cmcm space group, as shown in Figure 1B. Eight I atoms round the eight‐coordinated Cs^+^ ions in this configuration. The Cs─I bond lengths range from 3.92 to 4.18 Å. Four I^1−^ atoms and Cu^1+^ ions work together to generate a combination of edge‐sharing and corner‐sharing [CuI_4_]^3−^ tetrahedra.

The powder X‐ray diffraction (XRD) of the CsCu_2_I_3_ is shown in Figure 1C, and it is evident that all of the peaks fit the standard card (PDF#45‐0076) quite well. It was proved that the CsCu_2_I_3_ powder belongs to the orthogonal structure. GSAS software was used to structurally refine the XRD of the measured sample to have a deeper insight into the crystal structural information of CsCu_2_I_3_, based on the standard structure model of CsCu_2_I_3_ (JCPDS code 45‐0076). The predicted intensity is shown by the red line in Figure 1D, whereas the experimental test intensity is shown by black crosses. The discrepancy between the computed intensity and the experimental test intensity is shown by the blue line. The Bragg reflection position of the calculation model is indicated by the magenta vertical bar. Detailed refinement results are given in Table 1. Lattice parameters of CsCu_2_I_3_ are estimated to be *a* = 10.5357, *b* = 13.1652, and *c* = 6.0931 Å, respectively. The obtained values reveal that the *R_wp_* (weighted profile R factor) is 7.85%, the *R_p_* (profile residual, unweighted) is 5.96%, and *χ* ^2^ equal to 1.825. These findings suggest a high level of reliability in the refinement results.

**TABLE 1:** Refined crystallographic parameters of CsCu_2_I_3_.

Sample	CsCu_2_I_3_
Crystal system	Orthorhombic
Space group	*C m c m*
Lattice parameters	*a *= 10.5357(7) Å, *b *= 13.1652(7) Å *c* = 6.0931(4) Å, *α* = *β* = *γ* = 90°
Unit cell volume	*V* = 845.14(7) Å^3^
*R_p_*%	7.85
*R_wp_*%	11.53
*χ* ^2^	1.825

**FIGURE 2:**
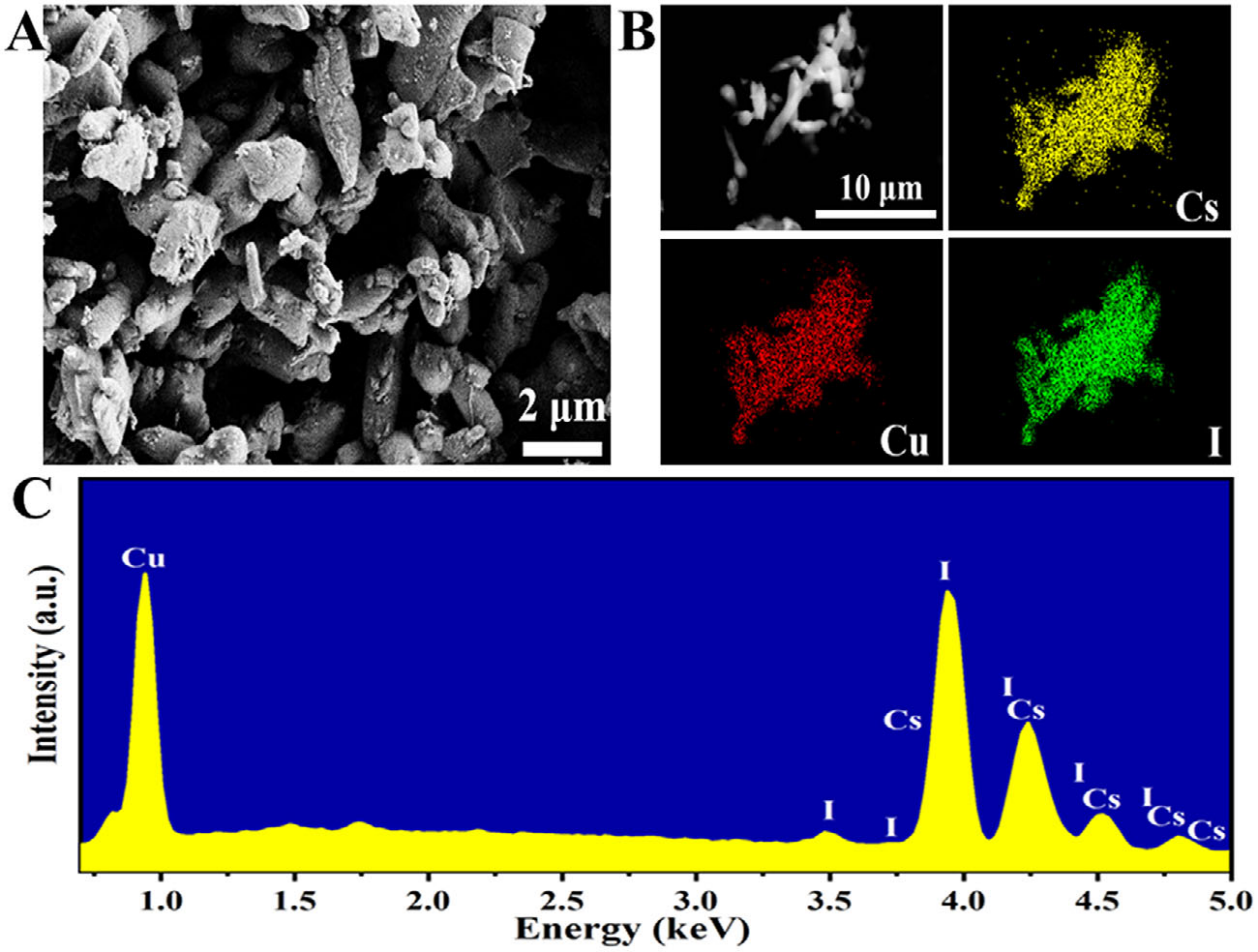
Morphology and element proportion characterization. (A) FE‐SEM image of CsCu_2_I_3_ powders. (B) Mapping of elements in CsCu_2_I_3_ powders. (C) EDS spectrum of CsCu_2_I_3_ powders.

The SEM images of CsCu_2_I_3_ phosphors were recorded to investigate the surface morphology and presented in Figure 2A. The SEM images show that the present phosphors have irregular shapes and their particle size was found to vary from several hundred nm to a few microns. Also, the element mapping results of the prepared CsCu_2_I_3_ powder are shown in Figure 2B. It is noticed that the Cs, Cu, and I elements are homogeneously dispersed over CsCu_2_I_3_ particles, conforming to the successful preparation of CsCu_2_I_3_ phosphor and the absence of a secondary phase. Further confirmation was made through the measured EDS spectrum (Figure 2C) and it shows the presence of Cs, Cu, and I elements in the prepared CsCu_2_I_3_ phosphors.

**FIGURE 3:**
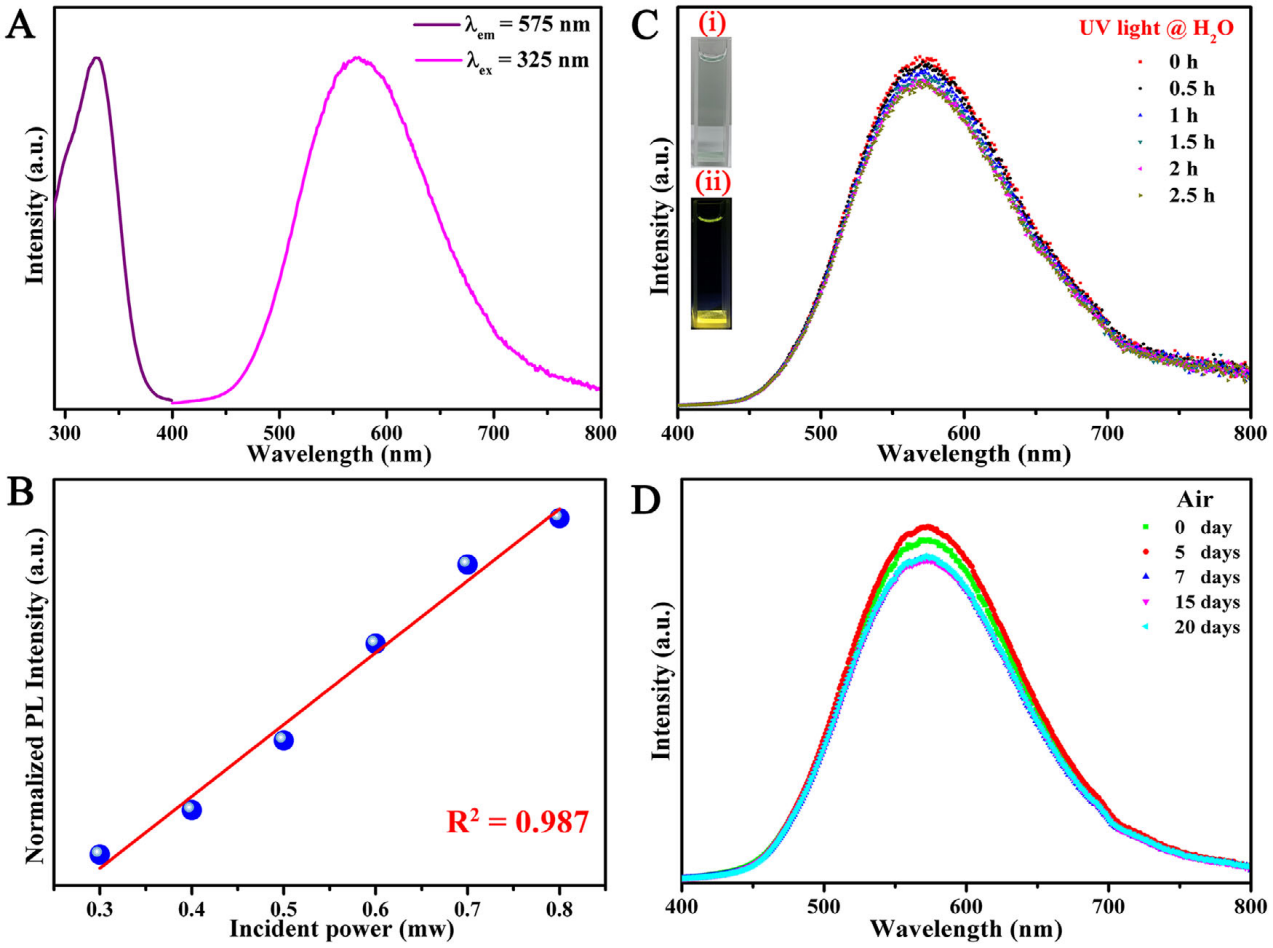
Room temperature optical properties of CsCu_2_I_3_ powder. (A) The room‐temperature excitation and emission spectra of CsCu_2_I_3_ powder. (B) Integrated broadband PL intensity of CsCu_2_I_3_ as a function of excitation power. (C) PL spectra of CsCu_2_I_3_ powder versus different soaking times under UV light. Inset shows optical images of the sample under (i) daylight and (ii) UV light in the deionized water. (D) PL stability versus different storage times in ambient air with RH of 35%.

The PLE and PL spectra of CsCu_2_I_3_ powder phosphors are shown in Figure 3A. When the detection wavelength is 575 nm, the PLE spectrum exhibited a wide spectrum, which indicates that the emitted radiation comes from a wide range of excitation wavelength excited states. It is important to remember that the PLE peak is in the deep ultraviolet and can be found at 325 nm. The emission spectrum is broad, with a peak at 575 nm, similar to the excitation the excitation spectrum. It can be seen from Figure 3A that there is a significant Stokes shift between the excitation spectrum and the emission spectrum. In general, the presence of permanent defect states in semiconductors can produce broadband emission. For this purpose, the emission intensity of CsCu_2_I_3_ by varying excitation power was measured. As shown in Figure 3B, power‐dependent PL measurements show linear behavior for CsCu_2_I_3_, which indicates that the broadband emission of CsCu_2_I_3_ does not originate from permanent defects. Therefore, the largely Stokes‐shifted broadband emission of CsCu_2_I_3_ was attributed to self‐trapping excitons (STEs). STEs are considered the formation of lattice defects by stimulated emission in metal halide, which can also be called transient lattice defects to some extent. For CsCu_2_I_3_, the [CuI_4_]^3–^ group was formed when Cu^+^ ions reside in tetrahedrally coordinated interstice formed by anion sub‐lattice (As shown in Figure 1B). The double [CuI_4_]^3−^ tetrahedra are stacked infinitely and share their edges to form [Cu_2_I_3_]^−^ anionic chains, and such structures are very similar to octahedral units. As we all know, octahedral structure is not stable, significant structural deformation occurs when the ground state is transformed into the excited state. Photogenerated excitons are easily trapped in distorted lattices, forming so‐called STEs. This elastic lattice distortion changes the nuclear coordinates, dissipates some of the exciton energy, and results in broadband emission with a large Stokes shift.^[^ ^23^ ^]^ The stability of metal halide materials has attracted much attention as well. Therefore, its PL stability and nature were also investigated by various experiments. As shown in Figure 3C, almost no fluorescence quenching occurs after immersion in water and ultraviolet irradiation for 2.5 h, and no changes in the spectral shape and peak position are observed. The two insets of Figure 3C show the photos of samples soaked in water under natural light and UV light. Under natural light, the sample is milky white, while under UV light, the sample emits bright yellow light. The CsCu_2_I_3_ powder emission performance of the CsCu_2_I_3_ powder can be sustained effectively even after 20 days of storage in ambient air for 20 days, as seen in Figure 3D. Therefore, CsCu_2_I_3_ exhibits good stability and maintains good fluorescence performance even if the sample is exposed to an extreme environment. This advantage makes it possible to apply CsCu_2_I_3_ powder for a long‐term wide application.

**FIGURE 4:**
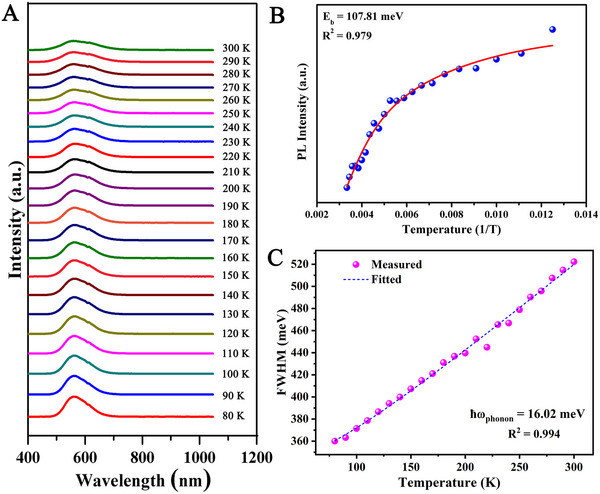
Variable temperature optical properties of CsCu_2_I_3_ powder. (A) PL spectrum of CsCu_2_I_3_ powder at varying temperatures. (B) The plot between PL intensity and temperature. (C) The plot of FWHM versus temperature (80–300 K).

The temperature‐dependent luminescence was measured (80–300 K) to study the exciton dynamics (see Figure 4A). As the temperature increases, a single emission peak is observed, which indicates that the yellow emission is produced by CsCu_2_I_3_ powder, and not by other impurities. It indicated that there was no phase change in the sample with the change in temperature. Figure 4B,C shows variations of emission intensity and FWHM (by Gaussian fit) with respect to temperature. The PL intensity versus temperature curve was fitted by using the equation as,^[^ ^24^, ^25^, ^26^ ^]^

(1) $$I_{T}=\frac{I_{0}}{1+A\exp\left(\frac{-E_{b}}{k_{B}T}\right)}$$

where, *k*_B_ and *E*_b_ are the Boltzmann constant and exciton binding energy, respectively. *I*(0K) and *I*(*T*) are the integrated emission intensities. The exciton binding energy of the studied phosphor is found as 107.81 meV (Figure 4B), which is higher than that of conventional perovskite materials.^[^ ^27^ ^]^ Such a large *E_b_* can ensure the generation of excitons at room temperature and promote their effective radiation recombination. Therefore, the sample exhibits a strong PL intensity. The interaction between phonon and electron can be obtained from the plot between FWHM and temperature (Figure 4C): *ћω* = 16.02 meV. It can be fitted by Equation (2)^[^ ^28^ ^]^

(2) $$FWHM\left(T\right)=2.36\sqrt{s}\hbar \omega_{phonon}\sqrt{\coth\frac{\hbar \omega_{phonon}}{2k_{B}T}}$$

The electron‐phonon coupling parameter S, the phonon frequency ℏω (where ℏ is the reduced Planck constant), and the Boltzmann constant *K_B_* are all represented in Equation (2). ℏω is found to be 16.02 meV by fitting Equation (2), indicating that the CsCu_2_I_3_ powder has a soft crystal structure that is advantageous to the creation of self‐trapped excitons (STE). This significant phonon‐electron interaction suggests that lattice vibration and exciton localization are closely related.^[^ ^29^ ^]^

**FIGURE 5:**
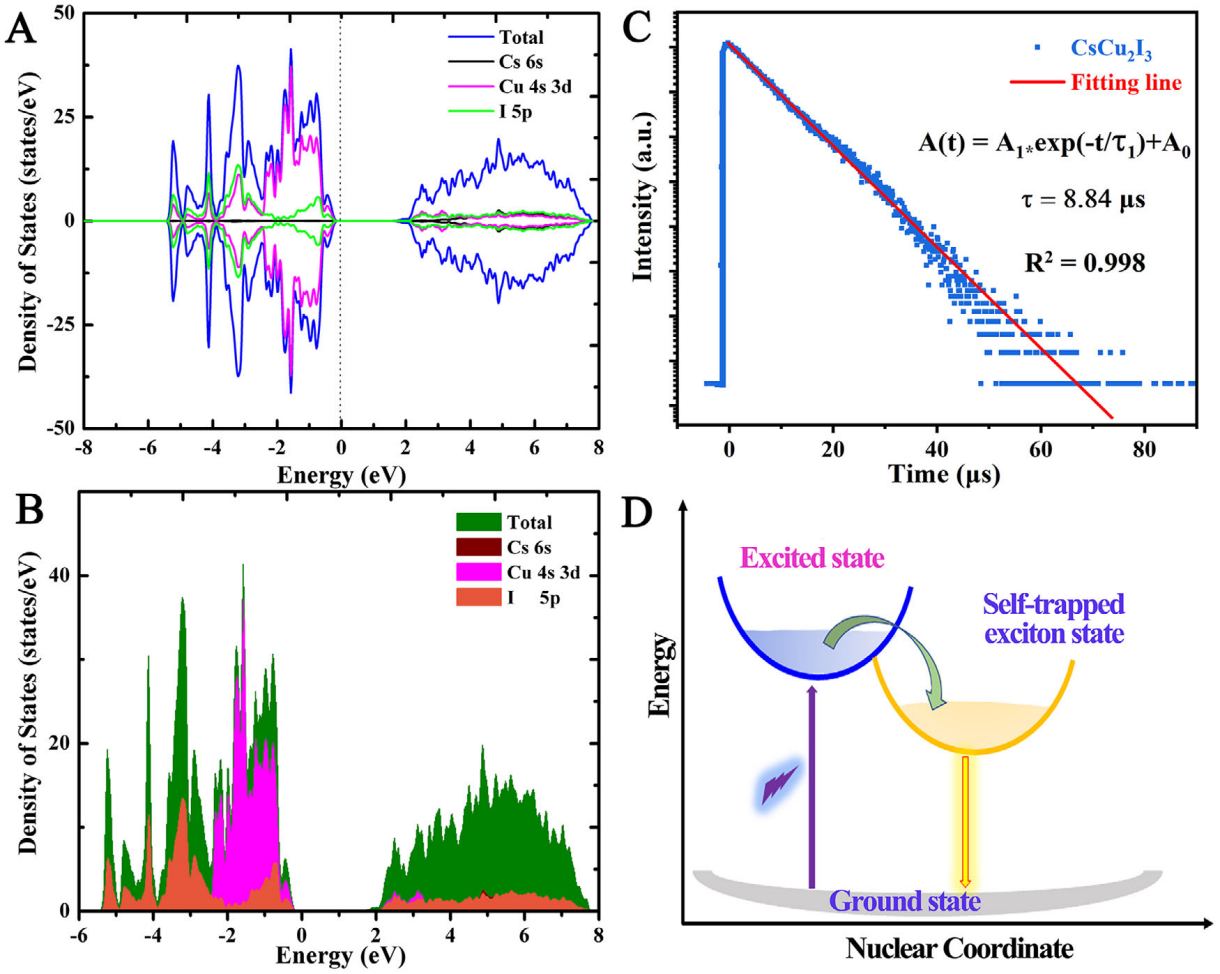
Electronic DOS and TRPL of CsCu_2_I_3_ powder. (A) Total electronic DOS for the CsCu_2_I_3_ powder. (B) Partial electronic DOS for the CsCu_2_I_3_ powder. (C) TRPL decay. (D) Configuration coordinate diagram of photophysical process.

Figure 5A,B shows the total and partial density of states (DOS) of the first‐principles calculation. Cu 3d and I 5p electrons make up the majority of the valence band maximum (VBM) in the state density, while Cu 4s and I 5p electrons make up the majority of the conduction band minimum (CBM). On the other hand, Cs^+^ ions have little contribution to VBM and CBM. The Cs^+^ ions can act as quasi‐isolating shells and the charge density concentrates in [Cu_2_I_3_]^−^ group and it is isolated by Cs^+^ in 1D direction. This one‐dimensional electronic structure illustrates a strong affinity for localization. The photo‐excited holes and electrons easily form STE and provide effective light emissions. The band gap calculated by the density of states is approximately 1.9 eV, which is not consistent with the experimental optical bandgap, because GGA‐PBE calculations underestimate the band gap.^[^ ^30^, ^31^, ^32^ ^]^

Figure 5C presents the time‐revolved PL decay profile of the CsCu_2_I_3_ powder. The lifetime values can be estimated by fitting the decay curve following the formula given by:^[^ ^33^, ^34^, ^35^ ^]^

(3) $$A\left(t\right)=A_{1}e^{\left(-t/\tau 1\right)}+A_{0}$$

The fluorescence lifetime was well fitted by a single‐exponential function and the value is 8.84 µs, which further proves that the fluorescence of the sample originates from the recombination radiation of STEs. Such long fluorescence lifetimes exist in most self‐trapping exciton luminescent perovskites such as Rb_2_CuBr_3_ and Cs_2_InBr_5_·H_2_O.^[^ ^36^, ^37^ ^]^ The relaxation mechanism is shown in Figure 5D for the explanation of decay time. The electrons in the ground state are forced rapidly to the excited states under photoexcitation. The electron in the excited state quickly relaxes to STEs due to the reorganization of the excited‐state structure. Finally, a long lifetime of 8.84 µs and a bright yellow light emission with a significant Stokes shift is produced by the recombination from self‐trapped exciton (STE) states to ground states. This finding is consistent with the luminescence spectra shown in Figure 3A.

**FIGURE 6:**
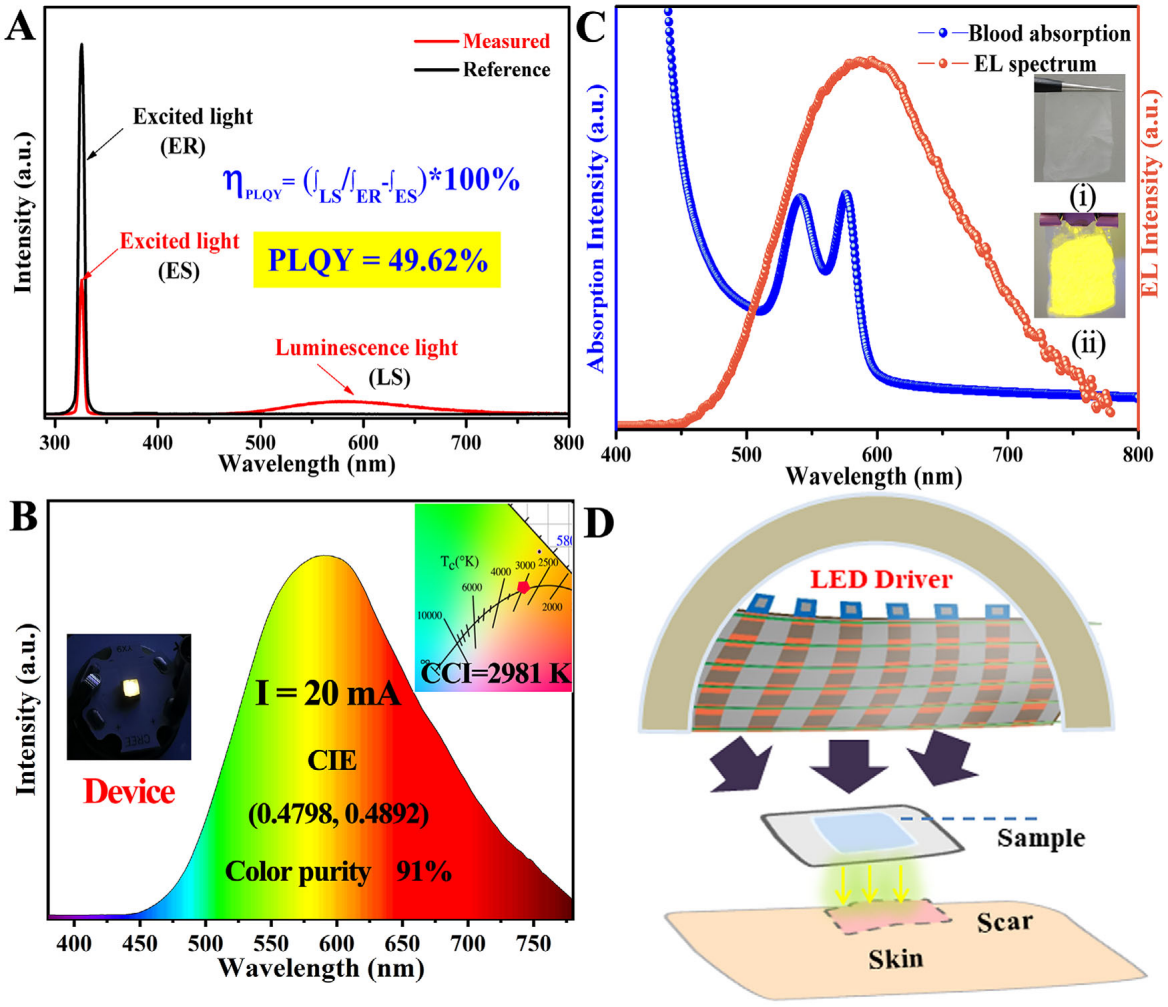
Performance of LED device. (A) Spectra of PLQY of CsCu_2_I_3_. (B) The EL spectrum of the fabricated LED device under a forward bias current of 20 mA. (C) Electroluminescence spectrum of LED device and the absorption spectrum of blood. (D) The LED therapy process.

In addition, we investigated the PLQYs of CsCu_2_I_3_ in Figure 6A. The PLQY of the yellow‐light CsCu_2_I_3_ powder is as high as 49.62%. Such a high PLQY is better than that of perovskite materials that emit yellow light (see Table 2). The inset of Figure 6A shows that the sample coated on the nonwoven emits bright yellow light under UV light irradiation. Finally, owing to its optical characteristics, high PLQY, nontoxicity, and excellent anti‐water behavior demonstrated above, we also carried out preliminary attempts to make LEDs based on CsCu_2_I_3_ powder. As illustrated in Figure 6B, a yellow LED device with the as‐synthesized phosphor emits a pure, bright yellow color at a current of 20 mA. The yellow emission peak at 575 nm in the EL spectrum is consistent with that of the PL spectrum. In addition, the CIE chromaticity diagram of the CsCu_2_I_3_ sample was calculated to be (*x*  =  0.4798, *y*  =  0.4982). Meanwhile, other important properties of general illumination sources are the correlated color temperature (CCT) and color rendering. Previous researchers have estimated color rendering indices and color temperatures for spectra obtained by combining different emitting light sources in white LED.^[^ ^47^, ^48^ ^]^ The prepared device has a CCT of 2981 K and a color purity of up to 92%, making it suitable for use as a warm white LED. Figure 6C shows the EL spectra and absorption spectra of blood. There is a tremendous overlap between the absorption spectrum of blood and the EL spectrum of CsCu_2_I_3_ pc‐LED, illustrating the possibility of the device as a yellow therapeutic lamp. Figure 6D shows the yellow LED therapy method. The UV chip irradiates the sample by emitting yellow light, and the yellow light irradiates the skin, allowing tissue repair and scar reduction. In combination with the waterproofing properties of the sample, the EL spectrum overlaps with the blood absorption spectrum, indicating that CsCu_2_I_3_ powder has potential applications in sunscreen skin care products and LED therapy.

**TABLE 2:** Comparison of PLQYs values of present phosphor and similar reported works.

Materials compositions	Morphology	Emission wavelength (nm)	PLQY (%)	Ref
CsPbBr_1.3_I_1.7_	Nanocrystal	556	40	[38]
CsPb_0.94_Mn_0.06_Cl_3_	Quantum dot	579	22	[39]
Cs_2_NaInCl_6_:Ag	Nanocrystal	535	31.1	[40]
Rb_7_Sb_3_Cl_16_	Powder	560	26	[41]
[DMEDA]PbCl_4_	1D	565	4.81	[42]
CsCu_2_I_3_	Nanorod	561	11	[43]
CsCu_2_I_3_	SC	≈583	6.5	[44]
CsCu_2_I_3_	Nanowire	542	–	[45]
CsCu_2_I_3_	SC	568	15.7	[30]
CsCu_2_I_3_	Thin film	548	20.6	[46]
CsCu_2_I_3_	Powder	575	49.62	This work

## 4 CONCLUSION

In conclusion, for the first time, all‐inorganic Pb‐free CsCu_2_I_3_ powder was synthesized in water using the water phase ultrasonic method. The STE state of CsCu_2_I_3_ gave a bright yellow PL emission with the highest PLQY of 49.62%, which is much higher than that reported for traditional yellow light‐emitting perovskite materials. Therefore, we produced monochrome UV‐pumped yellow LEDs with a CIE coordinate (0.4798, 0.4892) using the strong yellow emission of CsCu_2_I_3_. The mechanism of STE dynamics is further studied by TR‐PL measurements of CsCu_2_I_3_. Our work highlights the superiority of the synthesis method and provides a non‐toxic and earth‐abundant yellow all‐inorganic Pb‐free metal halide light emitter that is CsCu_2_I_3_, which is beneficial to the great progress of solid‐state lighting technology in the future and is expected to be used in the field of yellow LED therapy.

## CONFLICT OF INTEREST STATEMENT

The authors declare no conflicts of interest.

## Data Availability

The data that support the findings of this study are available from the corresponding author upon reasonable request.
